# Choices for cancer prevention for women with a *BRCA1* mutation? a personal view

**DOI:** 10.1186/s13053-023-00271-3

**Published:** 2023-11-29

**Authors:** Steven A. Narod

**Affiliations:** 1 Women’s College Research Institute, University of Toronto, 790 Bay Street, Toronto, ON Canada; 2 Dalla Lana School of Public Health, University of Toronto, Toronto, ON Canada

**Keywords:** Breast cancer, Ovarian cancer, BRCA1

## Abstract

With widespread testing for susceptibility genes, increasing numbers of women are being identified to carry a mutation in one of many genes which renders them susceptible to cancer. The first gene to be identified (in 1994) was *BRCA1* which increases a woman’s risk for breast cancer (70%) and ovarian cancer (40%). The prevalence of *BRCA1* gene mutations has been studied widely and in many countries, mostly in women affected with cancer. In many settings testing is offered routinely to women with serous ovarian cancer or early-onset or triple-negative breast cancer. It is preferable to identify a mutation in a healthy women prior to the diagnosis of cancer. The basic strategies for prevention include surgical prevention, chemoprevention and screening (early detection). Much progress has been made in the past two decades evaluating the benefits of these three approaches. In this commentary I provide my personal views regarding these various interventions in the context of counselling a newly diagnosed health woman with a BRCA1 mutation.

Women with a mutation (or pathogenic variant) in the *BRCA1* gene faces a lifetime risk of breast cancer of approximately 70% and a risk of ovarian cancer of about 40% [1]. The numbers of deaths from breast and ovarian cancer caused by *BRCA1* mutations are about the same. There are several tools available to reduce these risks, all have benefits but all have unwanted effects as well. Genetic testing for *BRCA1* mutations began in 1995 and over the past 27 years we have gained considerable knowledge about prevention by following women with *BRCA1* mutations and measuring the risks of cancer and death from cancer in these cohorts. Some participants in my international cohort study have been followed for 25 years [2].

Consider a 40 year old women who undergoes genetic testing in 2023 because her mother passed away from disseminated ovarian cancer at age 46. She discovers that she carries a pathogenic mutation in *BRCA1*. She has no past history of cancer and has both her ovaries and breasts intact. She is distraught by the news and arrives in my office with many questions “What are my risks? What are my options and how can I avoid the same fate as my mother?.

I tell her that there is a 70% chance she will develop breast cancer and a 40% chance of developing ovarian cancer by age 75 if she takes no action - but we can do much better. There are other cancer risks besides breast and ovarian cancer associated with *BRCA1* mutations [3], but these are rare and are not the subject of our discussion today. The risk estimate can be made more precise by incorporating additional measures such as personalised risks scores [4] or by consulting CanRisk [5], but this increases complexity it is not yet clear if these tools help guide decisions about cancer prevention. The most important factor is the current age of the patient.

Breast cancer is treatable if caught early, but most women who develop hereditary ovarian cancer will succumb to the disease. Screening for ovarian cancer by CA-125 and transvaginal ultrasound is not reliable; in our Polish study of women undergoing annual screening the majority of those with ovarian cancer detected eventually died of the disease [6].

We list the tools we have available for breast cancer prevention; there is chemoprevention (tamoxifen) and preventive surgery (bilateral risk-reducing mastectomy). Screening for breast cancer ideally consists of annual MRI examination (at age 40, our patient qualifies), mammography and ultrasound. For preventing ovarian cancer there are oral contraceptives [7] and preventive salpingo-oophorectomy [8]. She has not taken oral contraceptives before, but there is no data on the benefit of taking oral contraception in older women for the purpose of cancer prevention and I expect that few doctors would prescribe birth controls pills under these circumstances. There is a study underway looking at aspirin as chemoprevention [9]. There are no lifestyle factors which have been shown to modify the risk of breast cancer in BRCA1 carriers [10].

I recommend she undergo a baseline MRI breast examination. The baseline MRI will establish with a high level of certainty whether she has an occult breast cancer [11]. Occult breast cancers are not uncommon and are found in about 5% of asymptomatic *BRCA1* carriers [12]. MRI is a sensitive test [11], the prevalence of breast cancer found by pathological examination of preventive mastectomy specimens and by MRI is about the same [12]. If a cancer is discovered through MRI she becomes a *patient* (no longer a *previvor*) and she must be treated appropriately with chemotherapy and surgery. These topics are not covered further here.

If she is negative on the MRI examination, my next recommendation is for preventive salpingo-oophorectomy (BSO). This is now considered a *recommendation*, not merely an *option to consider*, based on 20 years of epidemiology research [13–15]. According to the current guidelines, the current recommendation for *BRCA1* carriers is age 35, but *BRCA2* carriers can delay the operation until age 45 [12–14] It is important that the fallopian tubes be removed as well as these may be the source of many of these cancers [16]. The beneficial effects of the salpingo-oophorectomy cannot be overestimated. We reported in 2014 that preventive BSO is associated with a 70% reduction in *all-cause* mortality [8]. We have recently updated this report and confirmed our earlier findings. If a woman at age 35 has a BSO her risk of dying of any cause to age 75 falls from 65% to 26% (Kotsopoulos et al., in press). There are side effects to be sure, such as those associated with early surgical menopause, but it is hard to argue with such a profound reduction in mortality. We saw an 83% reduction in the risk of dying of ovarian/fallopian/peritoneal cancer and a 50% reduction in the risk of dying of breast cancer after a salpingo-oophorectomy [8] The breast cancer effect is surprising and not easily explained, given that BSO does not reduce the risk of breast cancer [2, 17, 18] but reduces the case-fatality [19–21]. Furthermore, the beneficial effect of oophorectomy was present for women with ER-negative breast cancers as well as for post-menopausal women [20].

These numbers center our focus on the salpingo-oophorectomy. We estimate that for every three salpingo-oophorectomies performed we save one life. [This leads to an interesting paradox. The Bahamas is the country in the world with the highest prevalence of *BRCA1* mutations, 27% of breast cancer patients and up to one-half ovarian cancer patients carry a *BRCA* founder mutations [22]. On a recent trip to the Bahamas, I was told that, due to pandemic restrictions, elective surgeries including preventive salpingo-oophorectomies for carriers were put on hold and preference was given to providing debulking surgery for ovarian cancer patients. This is understandable from a moral point of view - we would not wish to withhold surgery from a cancer patient due to lack of OR space - but leads to a paradox. The majority of women with advanced ovarian cancer will succumb to their disease, in contrast the 39% reduction in mortality with BSO is profound and operating on *unaffected* women offers the potential to save many more lives].

Our patient goes for a bilateral salpingo-oophorectomy and hysterectomy (the hysterectomy is optional, but I think that if there is an opportunity to prevent new cases of cervical and endometrial cancer as well, then why not take it?) No cancer or precancerous lesion is found. She is prescribed estrogen only hormone therapy which has been shown to be safe for *BRCA1* carriers (i.e., does not increase the risk of breast cancer) [23] and estrogen relieves many of the symptoms of early menopause.

Now she faces a critical choice. Does she continue with the annual MRI examinations or opt for preventive mastectomy? After the BSO her risk of breast cancer remains high, but her risk of dying of breast cancer is lowered – perhaps to 5% to age 75. If she follows up with a bilateral mastectomy the risk falls further - to 1% or so. In a study from the Netherlands, eight of 722 women with a *BRCA1* mutation developed breast cancer after a bilateral mastectomy and one died [24]. In a Swedish registry-based study the mortality from breast cancer for *BRCA1* carriers after a preventive mastectomy remained about twice as high as the population norm [25].

How does this compare to MRI? In Ontario, we enrolled 489 women in an MRI prospective screening study, 91 developed breast cancer and four died of breast cancer [26]. We estimated the risk of death from breast cancer in a *BRCA* carrier undergoing MRI screening to be about twice that of the Ontario population. We estimated the probability of not dying of breast cancer at 20 years from the first MRI was 98.2%. A second Dutch group found that of approximately 400 *BRCA1* carriers followed by MRI, 33 developed breast cancer and five developed metastatic breast cancer [11]. It is clear that there will be many more breast cancers after MRI than after mastectomy, but it is not clear how the mortality rates compare. Perhaps it is too early to judge, given that it in screening studies it is first necessary to get breast cancer and then to die of it. Going forward, it is important that we consider BSO and annual MRI *in combination* if we are to propose an alternative to preventive mastectomy. And we need to follow the cohorts from age 35 to age 75. At present, I believe the combination of MRI and BSO offers the best alternative for patients who wish to avoid bilateral mastectomy and this needs to be discussed with the patient who chooses screening. They must know the numbers. The issue is goes beyond counting deaths, there other considerations as well, such as the operation itself, relief of anxiety and fear of cancer, the need for annual screening until age 70 (in our patient’s case, 30 future MRI examinations). Surgical options and type of reconstruction must be discussed. In general, women who opt for preventive surgery are happy with their decision and are relieved to know they have done everything they can to prevent cancer and can now avoid screening. Of course, many other women wish to retain their breasts. They must be aware that if they choose screening there is a high chance they will be eventually treated for breast cancer with chemotherapy and bilateral mastectomy.

In 1995, I had hoped that by 2022 preventive surgery would be replaced by a safe and effective means of chemoprevention. We are not there yet. Tamoxifen use is supported as part of chemoprevention recommendations [28, 29] but very few *BRCA1* carriers use it [29]. Part of the problem is the reluctance to take a pill daily that offers no sense of relief and there is no measurable indication that is working, Further, there is the notion that tamoxifen is not effective in preventing ER-negative cancers and the possible risk of endometrial cancer. Tamoxifen has been shown to be effective in preventing contralateral breast cancer in BRCA carriers [30, 31], but its benefit in preventing first primaries cancers remains to be seen. Our recent study showed a small but non-significant benefit of tamoxifen in primary prevention of breast cancer in BRCA1 and BRCA2 carriers [32].

The frontrunning candidate for cancer prevention in *BRCA1* carriers has switched from tamoxifen to denosumab. This drug targets the progesterone pathway rather than the estrogen pathway through blockage of the RANKL/RANK signaling pathway [33–36]. We have reported that exposure to progesterone (but not estrogen) is a risk factor for breast cancer after oophorectomy, so the progesterone pathway seems a logical target [23]. It has been shown to be efficacious in mouse models [33] and denosumab has the advantage of being administered as a subcutaneous injection every six months. One trial is underway but I think we should put an all-out effort into establishing if this candidate therapy has the potential to make preventive mastectomy obsolete.

Not too many years ago, patients would ask me “Why should I go for *BRCA1* testing if there is nothing I can do about it if I test positive?” A lot has changed since then and testing has become integrated in clinical oncology practice. The tools we have discussed above (preventive mastectomy, preventive salpingo-oophorectomy and MRI screening) have changed the landscape dramatically. Now our challenge is to get the tools in the right hands and to be available for as many women as possible. We need to widen our clinical criteria and increase our uptake of testing in order to identify unaffected carriers at a young age (preferably before age 40). If we rely on testing women with cancer first, we need to put more effort into getting their unaffected sisters and daughters involved. We need to overcome the barrier where the relative is not invited to be tested through the testing center, but is first contacted by the family member as direct contact has been shown to improve uptake [37]. In Canada we have developed TheScreenProject which gives the opportunity to have genetic testing for all Canadian men and women above age 18, regardless of personal history of family history of cancer [38]. We also need to facilitate the testing process where screening is offered widely at point of care to all (triple-negative or under 50) breast cancer patients and all ovarian cancer patients and then the genetic counsellor and geneticist are offered to consult with those with a positive test.

## Data Availability

Not applicable.
